# Hybrid Multi-Channel MAC Protocol for WBANs with Inter-WBAN Interference Mitigation

**DOI:** 10.3390/s18051373

**Published:** 2018-04-28

**Authors:** Thien Thi Thanh Le, Sangman Moh

**Affiliations:** Department of Computer Engineering, Chosun University, 309 Pilmun-daero, Dong-gu, Gwangju 61452, Korea; thienle@chosun.kr

**Keywords:** wireless body area network, multi-channel MAC, interference mitigation, throughput, priority, energy efficiency

## Abstract

Herein, we propose a hybrid multi-channel medium access control (HM-MAC) protocol for wireless body area networks (WBANs) that mitigates inter-WBAN interference significantly. In HM-MAC, a superframe consists of a random access phase and a scheduled access phase. That is, a carrier sensing multiple access with collision avoidance (CSMA/CA) phase and a time division multiple access (TDMA) phase are included in a superframe. The random access phase allows higher-priority users to transmit data packets with low latency and high reliability. The retransmission of data packets is also performed in the random access phase. The periodic data are transmitted in the scheduled phase, resulting in no contention and high reliability. A channel selection algorithm is also proposed to avoid collision between neighboring WBANs. The HM-MAC protocol allows multiple transmissions simultaneously on different channels, resulting in high throughput and low collision. The sensor nodes update idle channels by listening to the beacon signal; consequently, the sensor nodes can change the working channel to reduce inter-WBAN interference. According to our simulation results, HM-MAC achieves a higher packet delivery ratio and higher throughput with lower energy consumption than the conventional scheme in multi-WBAN scenarios. HM-MAC also causes lower end-to-end delays for higher-priority users.

## 1. Introduction

With developments in technology, wireless sensor nodes can be attached to the human body to monitor the vital signals of patients in hospitals or elders for medical purposes [1]. Currently, wireless body area networks (WBANs) are widely used in various applications in the medical field, personal healthcare, and movement detection [2]. For example, the blood pressure and heart beat rate of the human body can be sent to a heath monitoring center through a gateway called a coordinator, which is typically served by a smart phone. In real scenarios, when many people wearing a WBAN are within a small area such as a bus station or hospital, the wireless signals from the sensors to the coordinator in a WBAN may interfere with each other, resulting in the degradation of throughput [3,4]. In addition, signal loss can be caused by the movement of the human body. The mobility should be taken into account while designing the interference mitigation algorithm for WBANs [5]. In References [3,4], inter-WBAN interference has been shown to degrade the network performance in terms of packet delivery ratio and latency. Consequently, the interference mitigation and the associated medium access control (MAC) protocol have to be taken into design consideration to improve the network performance in WBANs.

The IEEE 802.15.4 [6] and 802.15.6 [7] standards define the physical and MAC layers, which are widely used as the base technology of WBANs. In the physical layer, the spectrum is divided into subchannels. Furthermore, to cooperate with the dense deployment network of WBANs, coexistence strategies have been considered in the IEEE 802.15.6 standard, which include beacon shifting, active superframe, and hopping channel strategies [7]. The hopping channel strategy allows a WBAN to avoid interference on the same channel by selecting a new channel according to the channel hopping sequence. The coordinator will generate a channel hopping sequence that is not being used by its neighbors. The channel hopping sequence is based on the maximum-length Galois linear feedback shift register [7] such that it is a random value. Because each WBAN performs data transmission in a single channel, the coordinator announces the new channel for the sensor nodes on the beacon signal. However, the new channel is taken by the coordinator without performing channels sensing, and may lead to a channel collision with another WBAN that selects the same channel. Currently, WBANs operate on a single channel MAC protocol for intra-WBAN transmission. The high-load traffic at the sensor nodes may cause collisions in intra-WBAN transmission, resulting in the degradation of network throughput. Currently, to cope with the need for higher network throughput, multi-channel MAC protocols are considered to increase the network throughput in various wireless networks such as wireless sensor networks, ad hoc networks, cognitive radio networks, and WBANs [8,9,10,11,12].

Motivated by the above mentioned challenges of inter-WBAN interference mitigation and multi-channel access, we propose a hybrid multi-channel MAC (HM-MAC) protocol to improve the network performance while mitigating the inter-WBAN interference. In the proposed protocol, a superframe consists of a carrier sensing multiple access with collision avoidance (CSMA/CA) phase and a time division multiple access (TDMA) phase. The CSMA/CA phase allows higher-priority users to transmit data packets with low latency and high reliability. The periodic data are transmitted in the TDMA phase, resulting in no contention and high reliability. The channel selection algorithm at the coordinator is also proposed to avoid collision between neighboring WBANs. The analysis and simulation results show that the proposed multi-channel MAC protocol allows many sensor nodes to transmit to the coordinator simultaneously on different channels, causing no collisions to neighboring WBANs. The higher-priority users using CSMA/CA achieve higher packet delivery ratio and lower delay than the low-priority users using TDMA. In addition, the sensor nodes listen to the beacon signal before switching to a new idle channel, which reduces the energy consumption of channel scanning.

The major contributions of our work in this paper are as follows:

- Hybrid medium access: The hybrid MAC superframe consists of the random access CSMA/CA phase and the scheduled TDMA phase. It is used to adapt to the transmission of different priority levels of traffic as well as allow the sensor to retransmit data packets.
- Framework of interference mitigation: This framework consists of inter-WBAN message exchange, channel selection, and intra-WBAN transmission. The inter-WBAN message exchange allows the coordinator to reserve idle channels for intra-WBAN transmissions by selecting different idle channels than its neighbors.
- Channel selection algorithm: A channel selection algorithm amongst neighboring WBANs is proposed, which allows each WBAN to select more than one channel for data transmission. We assume that the probability of selecting the data channel is the same in all WBANs in the network.
- Efficient use of multiple channels: To improve the network throughput, a coordinator will choose more than one channel for data transmission and one channel for control signal transmission. The multi-channel MAC improves the network throughput and reliability while avoiding the inter-WBAN interference and collision from neighboring WBANs.

In the following section, some related works are reviewed. In Section 3, the proposed HM-MAC protocol is presented with respect to the network model, interference mitigation, inter-WBAN communication and channel selection, and intra-WBAN communication. The theoretical analysis of throughput and delay are presented and discussed in Section 4. The performance of the proposed HM-MAC is evaluated via extensive computer simulations and compared to the conventional schemes in Section 5. Finally, Section 6 concludes the paper.

## 2. Related Works

In this section, we briefly review some current works that mitigate inter-WBAN interference. Recently, interference mitigation has been extensively investigated in multi-WBAN networks [13]. In real applications, inter-WBAN interference may occur when many WBANs try to access the same single channel simultaneously. The interference mitigation techniques can be categorized as a power control scheme, or medium access control schemes to schedule the transmission in the time domain or frequency domain [14,15,16,17,18,19,20]. In Reference [14], the power of the transmission link from the sensor nodes to the coordinator, or vice versa, are adjusted to achieve a high transmission rate while avoiding the interference from the neighbors. Another efficiency algorithm is to schedule the working nodes in the time domain or frequency domain, where the scheduling algorithms are applied to the WBAN such that the transmission of the WBAN is scheduled based on the node level [15,20] or the WBAN level [18,19]. However, in the scheduling algorithm, the coordinators of the WBAN have to negotiate for common scheduling, which results in increased latency.

Nevertheless, MAC protocols for WBANs have been widely studied to achieve better network performance of vital signals as well as to avoid inter-WBAN interference. Another research work [16] focuses on personal healthcare systems such as the aggregation system for a wearable network, which is formed by IEEE 802.15.4, IEEE 11073, and IEEE 802.11. The new architecture of the protocol stack connects many sensors with different IEEE standards in a data-centric point of view, in which cross-layer routing is focused in association with resource allocation and cooperative transmission. In Reference [17], the TDMA MAC protocol adapts the transmission of sensor nodes with regard to the transmission schedule and transmission duration, which aims to satisfy different quality of service requirements in terms of latency and energy consumption. In Reference [18], a MAC algorithm using CSMA or TDMA or a hybrid of CMSA and TDMA is used to avoid interference between sensor nodes within a single WBAN. In Reference [21], a MAC protocol reduced the collision in the multiple user environment while supporting the service quality of traffic such as vital signals. In Reference [22], TDMA is used to assign the time slot for the transmission from the sensor node according to the interference level. Another work [23] introduced the two-layer MAC protocol, in which the coordinator performs medium sensing using CSMA/CA; if the medium is idle, data transmission from the sensor nodes to the coordinator will occur. Consequently, the transmission within the WBAN will not collide with the other WBANs in the same vicinity. Another adaptive CSMA/CA technique is also applied to detect the idle medium at the coordinator in Reference [24], in which the coordinator applies the CSMA with the adaptive value of the contention window, which is obtained from historical values. The sensor nodes transmit data according to the schedule in the beacon signal received from the coordinator. The current MAC protocols apply the superframe with beacon, which contains the random access phase based on CMSA/CA and the schedule access phase based on TDMA to ensure signal continuity. Nevertheless, the existing MAC protocol allows intra-WBAN transmission in a single channel, which may lead to collision amongst the sensor nodes while accessing the medium.

To improve the bandwidth capacity and network performance, multi-channel MAC schemes have been applied to various wireless networks including WBANs. The multi-channel MAC protocol in Reference [10] is introduced for intra-WBAN transmission where the coordinator selects the idle channels before broadcasting the list of idle channels to its sensor nodes in the beacon signal. While receiving the beacon signal, the sensor nodes obtain the channel for transmitting data packets to the coordinator. In Reference [11], the coordinators use channel hopping techniques to allocate the transmission of sensor nodes into a specific channel and timeslot. Collisions with other neighboring WBANs as well as energy consumption are minimized. The channel hopping technique is applied to increase the coexistence capacity by allocating multiple WBANs into the same channel. Moreover, the WBAN transmission may be at risk under the cross-technology interference from other devices in a real scenario. In Reference [12], the joint of control routing and transmitting power is applied by selecting good-quality links to reduce interference at the WBAN. Furthermore, the cognitive radio technique is implemented in the WBAN to reduce interference, in which the coordinator of the WBAN detects the interference level in the working channel and switches to another channel [25].

In summary, MAC protocols can operate in a single channel or multiple channels to avoid intra-WBAN collisions. In addition, those MAC protocols also reduce inter-WBAN interference with the scheduling algorithm to allocate the transmission time and operating channel of each WBAN. On the contrary, some limitations are present in the existing works. First, the existing scheduling algorithm requires synchronization amongst WBANs that leads to long delays and high energy cost by transmitting negotiation messages. However, synchronization is a difficult task owing to the mobility of the WBAN in a real scenario. Furthermore, the current MAC protocols operate on a single channel, which cause collisions when accessing the medium. The density of nodes in the multiple WBANs will be increased to increase the interference. For example, the sensor node that transmits the emergency signal may collide with another signal from neighboring WBANs in the same channel. In this case, if two sensor nodes can operate in different channels, collisions will be reduced. Therefore, based on the limitation above, our work focuses on the MAC protocol operating on multiple channels while considering the interference signal from the other WBANs.

## 3. Hybrid Multi-Channel MAC

**Figure 1:**
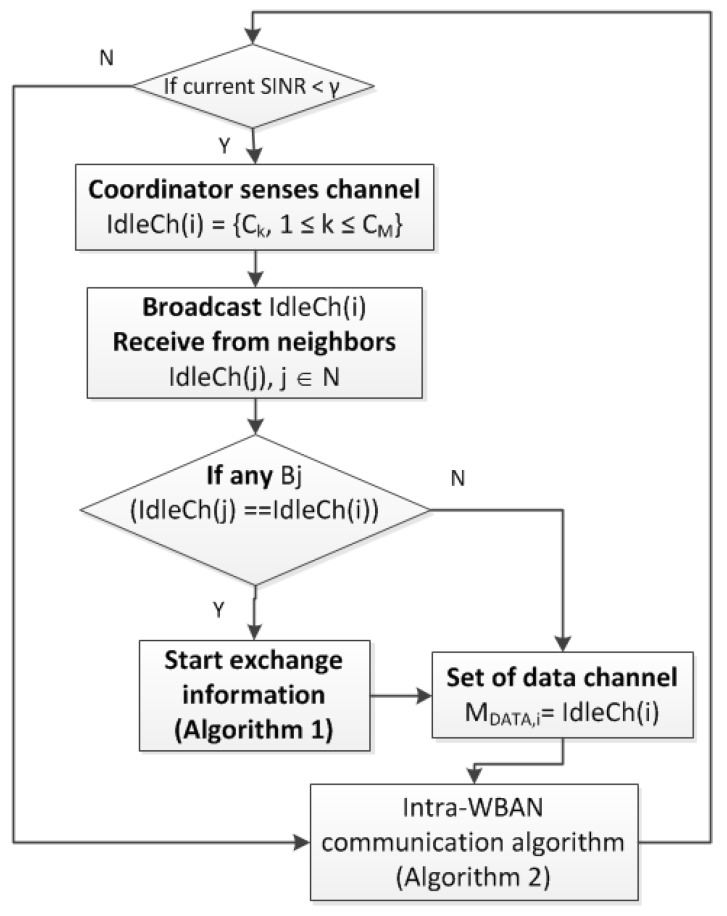
Framework of inter- wireless body area network (WBAN) interference mitigation for multi-channel medium access control (MAC).

In this section, we propose a hybrid multi-channel MAC with inter-WBAN interference mitigation, as shown in Figure 1. The framework of interference mitigation consists of inter-WBAN communication, channel selection, and intra-WBAN communication.

### 3.1. Network Model

The network consists of *N* WBANs, denoted as *B_i_*, 1 ≤ *i* ≤ *N*; each WBAN consists of one coordinator and *M* sensor nodes in which the sensor node is denoted as *s_ij_*, 1 ≤ *j* ≤ *M*. We assume that the sensor nodes of the WBAN can either transmit or receive data via multiple channels. For simplicity, the transmission range of each WBAN is set as a circle with radius *R*. We assume that the path-loss model for the intra-WBAN and inter-WBAN follows the free path-loss space model. The received power is calculated as:

(1) $$P_{rx}=\frac{P_{t}G_{t}G_{r}\lambda^{\eta }}{{\left(4\pi d\right)}^{\eta }}$$

The signal-to-interference-noise ratio (SINR) at the coordinator of *B_i_* is denoted as *γ_ij_* when any sensor node *s_i,j_* transmits data, and is calculated as follows:

(2) $$\gamma_{i,j}=\frac{P_{rx}(i,j)}{N_{0}+\sum_{\begin{array}{l} l\in (1,N)\\ l\neq j \end{array}}P_{rx}(i,l)}$$

where *P_rx_*(*i*, *j*) is the received power at *B_i_* from sensor node *s_i,j_*; *P_rx_*(*i*, *l*) is the received power at *B_i_* from the neighboring *B_l_*. The SINR threshold value is denoted as γ*_th_*; the coordinator adds the sensor node *s_i,j_* into the list of interfered nodes if γ*_i,j_* < γ*_th_*. If the coordinator detects any interfered nodes, the coordinator will start the interference mitigation algorithm.

We consider *N* WBANs working on a spectrum of *K* channels, denoted as *C_k_*, 1 ≤ *k* ≤ *K*. Among the *K* channels, a common channel exists for inter-WBAN messages exchange and *K −* 1 channels for intra-WBAN transmissions. For the intra-WBAN, each WBAN occupies one control channel and *M_DATA_* data channels, which can be explained as follows: The control channel is used for the transmission beacon and acknowledgement signal from the coordinator to the sensor nodes, while the data channel is used for data transmission from the sensor nodes to the coordinator. The relationship matrix between WBANs and channels is represented as:

(3) $$M_{DATA}=\left[\begin{array}{ccc} M_{1,1} & M_{1,k} & M_{1,K}\\ M_{i,1} & M_{i,k} & M_{i,K}\\ M_{N,1} & M_{N,k} & M_{N,K} \end{array}\right]$$

where the rows represent the WBAN index and the columns represent the channel index. The value of *M_i,k_* is set to 1 if *B_i_* uses channel *C_k_*; otherwise, *M_i,k_* is set to 0.

When *B_i_* is interfered, as in (2), we call the neighboring WBAN as the source of interference. The neighboring WBAN can either transmit or receive data simultaneously over the same frequency channel or when the neighboring WBAN transmission is in the transmission range of *B_i_*. We call *B_j_* as the one-hop neighbor of *B_i_* if the transmission range of *B_j_* and *B_i_* overlaps. Therefore, the interference condition can be obtained as *I_i,j_* = 1 if *d_i,j_* < R. Owing to the overlapped transmission range, if the signal from *B_i_* can be detected at *B_j_*, it indicates that the inter-WBAN interference occurs at the specific time and channel. The interference matrix is a matrix that identifies the interfered WBAN, where *I_i,j_* = 1 denotes the interference between the WBAN *B_i_* and *B_j_*, as follows:

(4) $$I_{}=\left[\begin{array}{ccc} I_{1,1} & I_{1,j} & I_{1,N}\\ I_{i,1} & I_{i,j} & I_{i,N}\\ I_{N,1} & I_{N,j} & I_{N,N} \end{array}\right]$$

We define a condition to avoid the interference between two nearby WBANs as follows: {*M_DATA_*_(*j*,:)_ ∩ *M_DATA_*_(*i*,:)_ = Ø| *I_i,j_* = 1}. We define a common channel for inter-WBAN transmission as follows: the coordinator of WBANs can communicate through the first channel of set *C_k_.* The intra-WBAN channel set is defined as follows: *M* sensor nodes can transmit data to the coordinator through *C_M_* channels without collision, *C_M_* ≤ *M.*

We assume that the energy level of the sensor nodes is lower than that of the coordinator. Therefore, the channel sense is obtained only at the coordinator using the energy level detection. The coordinator applies the channel sense using the energy level detection. The channel *C_k_* is idle if the energy level detection is above a threshold *E_Thr._*

### 3.2. Interference Mitigation

**Figure 2:**
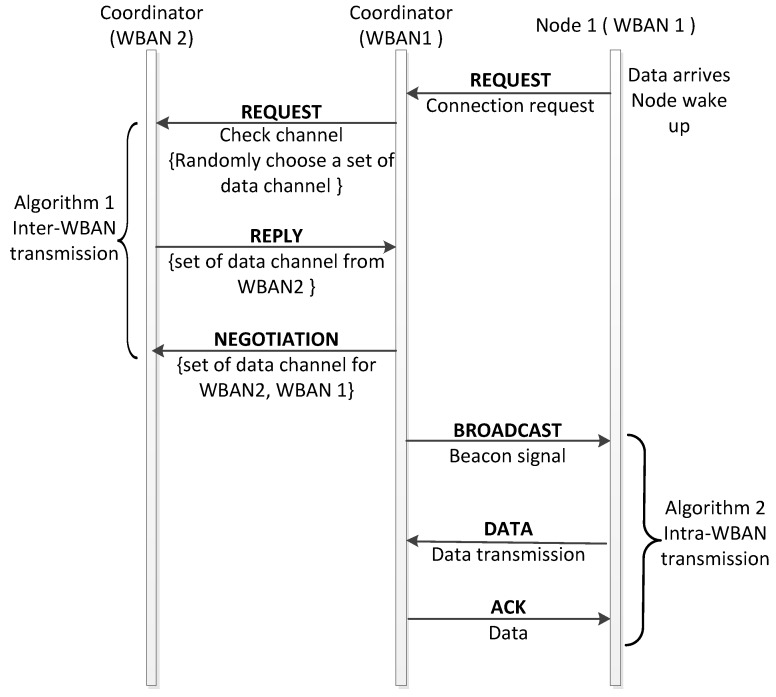
Inter-WBAN communication.

We developed a framework for an interference mitigation algorithm that supports multiple WBANs, as shown in Figure 1, which can be explained as follows. The coordinator will list the interfered sensor nodes using the SINR value. Subsequently, the coordinator will sense the idle channel by the energy level detection. Next, the coordinator will exchange the list of idle channels to its one-hop neighbors, as shown in Figure 2. The received message from the one-hop neighbors contains the list of idle channels from the neighbors. If there exists any neighbor *B_j_* who has the same idle channel set as *B_i_*, then *B_i_* and *B_j_* will exchange messages to find a reasonable data channel set as in Algorithm 1. The total available idle channels at two WBANs is called *TotalAvaiCh_i,j_* = *IdleCh*(*i*) ∩ *IdleCh*(*j*). When a WBAN finds its own data channel set that does not interfere with the neighbor, intra-WBAN communication starts, as given in Algorithm 2.

**Algorithm 1.** Channel selection algorithm.**Input: A** set of available channels at neighbor *Bj* (*IdleCh*(*j*), *j*
*Є NI , M_DATA,i_* = Ø)**Output:** A set of data channels for each WBAN {*M_DATA,i_* }1. **Step 1.** Calculate the total available channels for *Bj* and *Bi*:2.  *TotalAvaiCh* = *IdleCh*(*i*) ∪ *IdleCh*(*j*)3. **Step 2.** Calculate the priority value for each WBAN as in (5)4. **Step 3.** The number of channels for WBAN(*i*) in *TotalAvaiCh* with a higher *PVal_i_* calculated as follows5. **If**
*TotalAvaiCh* > Δ*C_i_*6.  *x* = max([*TotalAvaiCh*/2], Δ*C_i_*)7. **else**8.  *x* = min([*TotalAvaiCh*/2], Δ*C_i_*)9. **end if**10. **Step 4.** The number of channels for the other WBAN is calculated as follows11.  y=|TotalAvaiCh|−x12. **Step 5**. The intra-WBAN channel set at a WBAN13. **for**
*cnt* = 1: *x*14.  *M_DATA,i_* = *M_DATA,I_* ∪ *TotalAvaiCh*(*cnt*)15. **end for**16.  *M_DATA,j_* = *TotalAvaiCh* ∩ *M_DATA,i_*

### 3.3. Inter-WBAN Communication and Channel Selection

The inter-WBAN communication process is taken amongst WBANs with the same available idle channels. The inter-WBAN communication with the exchanging message steps is as shown in Figure 2. The coordinators will send the “*REQCh*” message that contains the set of *IdleCh* to its one-hop neighbors. The coordinator also waits for the “*REP*” messages from its neighbors, which contains the set of *IdleCh* and the priority value as in (5). Upon receiving the “*REP*” message from the one-hop neighbors, the WBAN coordinator will reselect its data channels by running Algorithm 1. After selecting the data channels, the coordinators will again broadcast the “*LISTCh*” message, which contains the list of data channels for each WBAN. Consequently, the channels in *TotalAvaiCh* will be occupied by two neighbors without interference. 

In Algorithm 1, the coordinator creates a set of total available channels as in step 1. Subsequently, to verify the interference level at the WBAN, each WBAN will calculate the priority value, which considers the operating time and data channel set as follows:

(5) $$Pval_{i}=\frac{\Delta C_{i}}{C_{\max}}\times \frac{\Delta TI_{i}}{\Delta T}$$

where Δ*T* is the total operating time calculated by the number of superframes; Δ*TI_i_* is the number of interfered superframes in which the WBAN is interfered with by other WBANs; Δ*C_i_* is the average channel for the intra-WBAN communication during Δ*T*; Δ*C_max_* is the total available channel. 

In Algorithm 1, the total available idle channel for *B_i_* and *B_j_* is calculated as the joint of *IdleCh*(*i*) and *IdleCh*(*j*) in step 1. In step 2, the priority at each WBAN will be calculated as in (5). In step 3, the WBAN with a higher *Pval* will have a higher priority to access the total available idle channels, or the WBAN will obtain more idle channels than the WBAN with a low *Pval*. At the WBAN *B_i_* with a higher *Pval*, the number of *TotalAvaiCh* is compared to the average channel Δ*C_i_*, and then the number of channels for *B_i_* is calculated as in Algorithm 1. The other WBANs will obtain the channel as in step 4. Finally, the data channel for each WBAN is calculated in step 5.

The separate channel is defined as in IEEE 802.15.6 [7], which is three channels to avoid interference from adjacent channels. To avoid the collision in the time domain as well as synchronization, the coordinators will send a beacon signal via the control channel as follows: Each coordinator will sense the idle channel before transmitting its beacon signal. The sensor node will stay awake to listen to the beacon signal of its WBAN. After receiving the beacon signal, the sensor nodes have the knowledge of the data channel and the TDMA schedule; subsequently, it switches to the data channel and waits until its time slot. The radio will be turned off after the transmission. At the end of data transmission, the sensor node turns on at the control channel to listen to the acknowledgement and the next beacon. Therefore, it reduces the energy from the listening channel and sensing channel. This algorithm provides a strategy to support channel switching from the control channel to the data channel at the sensor nodes and the coordinator.

### 3.4. Intra-WBAN Communication

We assume that the priority of the data generated at each sensor node can be categorized as in the IEEE 802.15.6 standard. The sensor nodes have knowledge of the *C_M_* data channel by receiving the beacon signal, 1 ≤ *C_M_* ≤ *M*. Assuming that a packet is always present in the buffer at each sensor node, if the packet fails to transmit to the coordinator, it will be retransmitted in the future. The sensor nodes will contend the idle channel for packet transmission using CSMA/CA with a specific *CW.* We also use the *CW_min_* and *CW_max_* as defined in the IEEE 802.15.6 standard. Here, we define *CW_avg_* as the average *CW* of the node during the operating time Δ*T*. At each sensor node, the new value of *CW* is calculated according to the average *CW_avg_* as follows:

(6) $$CW_{new}=\{\begin{array}{cc} \max(CW_{avg},CW_{\max}/2) & if\\ \min(CW_{\max},CW_{avg}) & otherwise \end{array}\begin{array}{c} CW_{avg}<CW_{\max}/2 \end{array}$$

**Figure 3:**
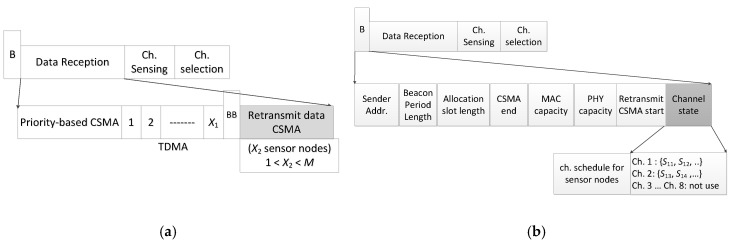
Hybrid multi-channel medium access control (HM-MAC) superframe: (**a**) intra-WBAN transmission; (**b**) beacon signal.

The superframe for intra-WBAN transmission is shown in Figure 3a. We modified the superframe of the IEEE 802.15.6 standard using two beacon signals, the priority-based CSMA, the TDMA part, and the retransmitted CSMA part. In our superframe, the first beacon signal is sent by the coordinator at the beginning of the superframe, which includes the control signal as shown in Figure 3b. The coordinator will broadcast the list of data channels in the field “*Channel_state*” in the beacon signal to the sensor nodes as well as the list of channels that the WBAN cannot use. In the data reception part, we use a hybrid medium access technique for both CSMA and TDMA. In the first CSMA part, only the high-priority users can access the channel. The other sensor node will access the channel in the TDMA part. In case of lost packets from sensor nodes, the coordinator will broadcast a second beacon called *BB* after the TDMA part. The second beacon signal consists of the list of sensor nodes that need to retransmit as well as the length of transmission.

The TDMA transmission is scheduled in the superframe after the transmission length of the priority-based CSMA. Consequently, the high-priority nodes can switch the channel to transmit in the priority-based CSMA portion after receiving the beacon, while the low-priority nodes have to wait until the end of the CSMA portion. In Algorithm 2, the CSMA protocol for the intra-WBAN transmission algorithm is shown. In the first step (Step 1), each node listens to the channel and receives the beacon signal from the coordinator, and then calculates the value of *CW* and *backoff_counter*. The highest-priority node will obtain the channel as in Step 2. The *backoff_counter* is reduced when the channel is busy. In Step 3, the low-priority node will transmit data in the allocated slots in a specific channel that is assigned by the coordinator. In case of a failed transmission, the node will wait for the second beacon signal, and repeat Step 2 in the retransmitted CSMA part.

**Algorithm 2**. Intra-WBAN communication algorithm.**Input: A** set of data channel *M_DATA_***Output:** A set of data channel for sensor nodes (by CSMA/CA)  **Step 1.**1. **for** each nodes2.  receive Beacon3.  obtain the value of *CW*, *backoff_counter*4. **end for**  **Step 2.**5. **if** the priority of node is highest6.  compare the current channel (*Ci*) to the set of data channel7.   **if**
*Ci* belongs to *M_DATA_*, continue8.   **else,**9.    pick up a random channel (*Cr*) in *M_DATA_*,10.    **if**
*Cr* is idle, decrease *backoff_counter*11.     **if**
*backoff_counter* is zero, transmit data to coordinator12.     **else**, lock *backoff_counter*13.     **end if**14.    **else,** choose another *CW* and pick up another channel15.    **end if**16.   **end if**  **Step 3**. // the priority of node is not the highest17. **else**18.  pick up a channel (*Cr*) for TDMA transmission in *M_DATA_*, which is broadcasted in the beacon signal19.  wait until its allocated time slot, transmit data to coordinator20. **end if**

### 3.5. Multi-Channel Multi-WBAN Example

**Figure 4:**
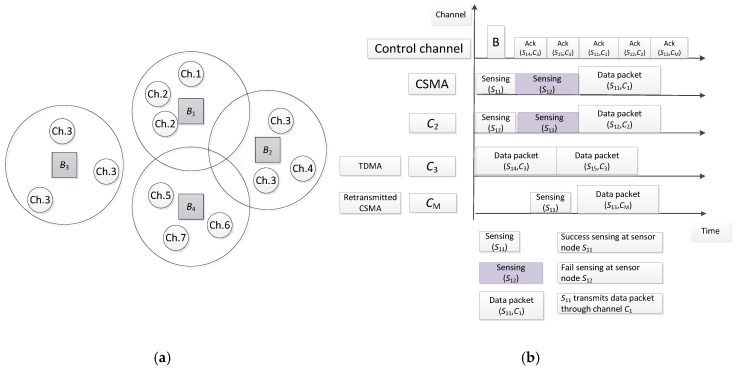
Multi-channel example: (**a**) network configuration; (**b**) intra-WBAN transmission.

An example of multi-channel multi-WBAN configuration is shown in Figure 4a. Assume that four WBANs exist, in which each WBAN has one coordinator and several sensor nodes. The number written in the small circle indicates the index of the data channel. In Figure 4a, B1, B2, and B4 share the same vicinity with the set of channel TotalAvaiCh = {1, 2, 3, 4, 5, 6, 7}, while B3 is not interfered with any WBANs, such that B3 can reuse channel {3} for intra-WBAN transmissions.

The example in Figure 4b shows intra-WBAN transmission. The high-priority nodes {S11, S12, S13} sense channels C1 and C2; if there is no collision, the data packets are transmitted to the coordinator. Channel C3 is divided into slots for TDMA transmission at the sensor node {S14, S15}. The sensor node {S13} will retransmit data through channel CM because of failure during priority-based CSMA. The acknowledgement of the successful transmission at the coordinator will be transmitted at the control channel.

## 4. Throughput and Delay Analysis

### 4.1. Probability of Successful Transmission

Throughput is measured by the number of successfully received packets by the given time. Assume that *N* WBANs exist in the network, and each WBAN consists of *M* sensor nodes and one coordinator. The total number of channels for the network is *K* data channels, 1 ≤ *K* < *N*, in which each channel is divided into *T* data time slots and the total generated packets during *T* time slots is *G* packets.

The number of successfully received packets is calculated as:

(7) $$P_{s}=G\times p_{s}$$

where *p_s_* is the probability that a packet is successfully received at the coordinator.

In case of an intra-WBAN transmission, at channel *C_k_*, data collision occurs when two sensors simultaneously transmit packets in *C_k_* at slot *t_i_*. Let *p_s,i_* be the probability of successful transmission at slot *t_i_* when *C_k_* is idle. As such, *p_s,i_* is given by:

(8) $$p_{s,i}^{\left(k\right)}=p_{idle}^{\left(k\right)}\cdot p_{s}(t_{i})$$

where *p_s_*(*t_i_*) is the probability of successful transmission in slot *t_i_*. The Bernoulli trial will be given to represent *p_s_*(*t_i_*) when another node tries to transmit packets. We represent *p_s_*(*t_i_*) as follows:

(9) $$p_{s}(t_{i})=p_{i}\left(\begin{array}{l} M\\ 1 \end{array}\right)q{\left(1-q\right)}^{M-1}$$

where *q* is the probability that a node performs successful channel sensing, and *p_i_* is the probability that a packet will transmit in time slot *t_i_*; *q* and *p_i_* are shown as:

(10) $$q=\frac{1}{\bar{CW}}$$

where $\bar{CW}$ is the average value of the contention window, and $p_{i}=\frac{1}{T}$.

The probability of successful transmission in *C_k_* is given as:

(11) $$p_{s}^{\left(k\right)}=\sum_{i=1}^{T}p_{s,i}^{\left(k\right)}=\sum_{i=1}^{T}p_{idle}^{\left(k\right)}p_{s}(t_{i})=\sum_{i=1}^{T}p_{idle}^{\left(k\right)}p_{i}\left(\begin{array}{l} M\\ 1 \end{array}\right)q{\left(1-q\right)}^{M-1}$$

The interference area between two nearby WBANs {*B_i_* and *B_j_*} is denoted as *L_i,j,_* and locates the set of interfering sensor nodes of *B_i_* and *B_j_* as {*S_i_*} and {*S_j_*}, respectively. In addition, the data channel sets of *B_i_* and *B_j_* are given as {*C_i_*} and {*C_j_*}, respectively. To calculate the probability that channel *C_k_* is idle or $p_{idle}^{\left(k\right)}$, we also assume that the probability of selecting the channel is equal. In addition, only the interference in the same channel is considered herein, such that the condition of interference is {*C_i_*} ∩ {*C_j_*} = *C_k_*. If *B_i_* selects channel *C_k_*, the probability that *B_j_* selects a random channel is 1/*K*, and the (*K*-1)/*K* represents the probability of selecting a channel not chosen by *B_i_*. The probability that two WBANs will select the same channel is given by:

(12) $$p_{col}^{\left(k\right)}=1-\frac{K}{K}\frac{K-1}{K}=1-\frac{K-1}{K}$$

Hence, the probability that *C_k_* is idle is given by:

(13) $$p_{idle}=p_{idle}^{\left(k\right)}=1-p_{col}^{\left(k\right)}=\frac{K-1}{K}$$

Therefore, the probability of successful transmission over *K* channels is represented as:

(14) $$p_{s}=\sum_{k=1}^{K}p_{s}^{\left(k\right)}$$

### 4.2. Transmission Time and Throughput

Transmission time is defined as the total time to transmit a packet including sensing time *T_s_*, time to transmit a data packet *T_data_*, time to receive acknowledgement *T_ack_*, time to receive beacon *T_B_* and *T_B2_*, and delay due to retransmission *T_d,R_*. Thus, the transmission time *T_i_* of packet *i*^th^ is given as:

(15) $$T_{i}=T_{s}+T_{data}+T_{ack}+T_{B}+T_{B2}+T_{d,R}$$

The sensing time *T_s_* is the duration that a sensor node performs the sensing of an idle channel. We divide *T_s_* into two cases as follows. In case (1), if the sensor node performs as an idle channel, it will transmit data, such that *T_s_* is calculated as:

(16) $$T_{s}=T_{bo}N_{bo}p_{s,i}^{k}$$

In case (2), if more than two nodes perform at the same channel, the sensor node will lock the counter at a random time *T_rand_*, which takes the random values between [0, Tdata] and performs sensing on another channel, such that *T_s_* is given as:

(17) $$T_{s}=(1-p_{s,i}^{k})T_{rand}+T_{data}$$

The delay due to retransmission *T_d,R_* is simply given when a packet is transmitted in the Retransmitted Data part as in Figure 3a. Thus, the delay due to retransmission is given by:

(18) $$T_{d,R}=T_{W}+T_{data}$$

where *T_W_* is the duration of the priority-based CSMA and TDMA part in the superframe.

Therefore, the total transmission delay is calculated as:

(19) $$T_{D}=\sum_{i=1}^{G}T_{i}$$

The throughput at a WBAN is a fraction of the successfully received packets at the coordinator to the total transmission delay, which is given as:

(20) $$Throughput=\frac{P_{s}}{T_{D}}$$

We analyze the throughput per node and the delay per node in the multi-WBAN environment, where each WBAN operates on multiple channels. The number of channels per WBAN is varied from one to four, so that the sensor nodes can switch to the idle channel. Each WBAN consists of one coordinator and 10 sensor nodes, assuming that each sensor node generates a random number of packets in each superframe. Each packet is 70 bytes long and the duration of a superframe is 100 ms. In order to extract the successfully received packets from the sensor node, we vary the probability of interference per channel from 0.1 to 1.

**Figure 5:**
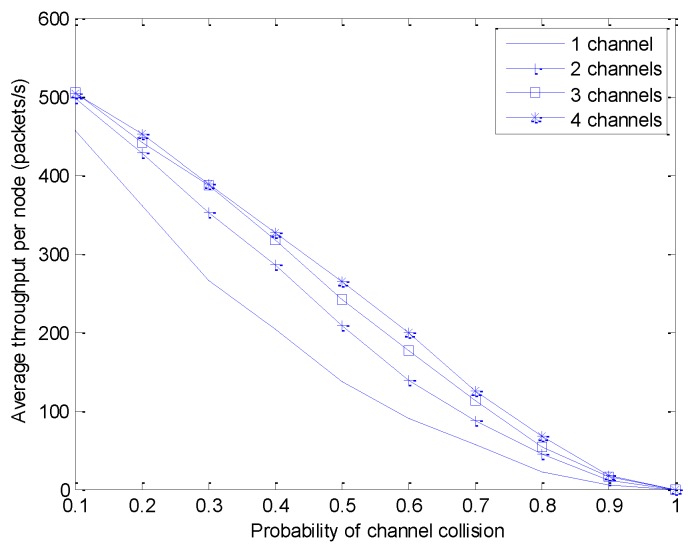
Average throughput per node.

Figure 5 shows the analysis of average throughput per node in four cases of multiple channels per WBAN. The analysis results show that the average throughput declines with the increased probability of channel collision. By using multiple channels per WBAN, however, the average throughput slightly increases compared with the single-channel MAC protocol because the sensor node can switch to another channel for data transmission.

**Figure 6:**
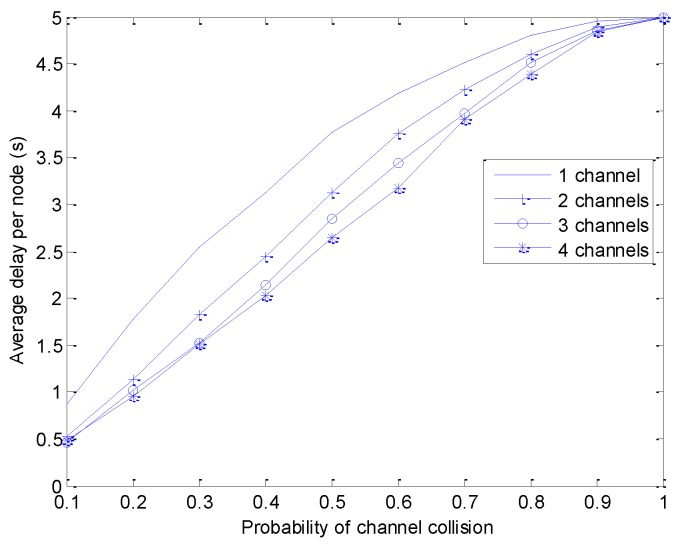
Average end-to-end delay.

Figure 6 shows the analysis of average end-to-end delay at each node in four cases of multiple channels per WBAN. It can be observed that the average delay increases as the probability of channel collision is increased. As shown in the throughput analysis, the average delay is also improved in case multiple channels are used for data transmission.

### 4.3. Channel Utilization

Channel utilization is defined as the average bandwidth for successful transmission to the given bandwidth. The average bandwidth for the transmission of the WBAN *B_n_* is given as:

(21) $$E_{BW,n}=W\cdot E_{idle,n}$$

where *W* is the bandwidth of the channel and *E_idle,n_* provides the average number of idle channels that a WBAN uses for data transmission in a given *T.*

The probability of *x* channels with successful transmissions in *K* channels is denoted as:

(22) $$p_{idle}=\left(\begin{array}{l} K\\ x \end{array}\right){\left(p_{idle}^{}\right)}^{x}{\left(1-p_{idle}^{}\right)}^{K-x}$$

The average number of idle channels at the WBAN *B_n_* is calculated as follows:

(23) $$E_{idle,n}=\sum_{k=1}^{K}p_{idle}^{k}$$

Therefore, the channel utilization at the WBAN *B_n_* is given as:

(24) $$\omega_{n}=\frac{E_{BW,n}}{K\cdot W}$$

## 5. Performance Evaluation

In this section, the performance of the proposed HM-MAC is evaluated via extensive computer simulations using OMNET++ and then compared to that of conventional protocols. In the recent literature, most multi-channel MAC protocols mainly focus on the multi-channel TDMA MAC, in which the intra-WBAN transmission operates on a single channel. The conventional multi-channel MAC, called MC-MAC [10], is selected for performance comparison because MC-MAC considers the multiple channels while using the beacon mode with the superframe.

### 5.1. Simulation Environment

The network consists of *N* WBANs and *C* channels. In each WBAN, *M* sensor nodes exist. We assume that the total number of channels is 12 and each WBAN consists of one coordinator and 10 nodes. In each WBAN, we deploy four channels for intra-WBAN transmission [10]. The simulation parameters are summarized in Table 1. To evaluate the network performance, we aim to derive the packet delivery ratio, network throughput, latency, and energy consumption at each WBAN with respect to the network density. The network density is chosen as the number of neighboring WBANs that share the same vicinity. In HM-MAC, we assume that the traffic priority is categorized as in the IEEE standard 802.15.6 [7]. If the nodes carry vital data such as an emergency, they are considered as high-priority traffic nodes that will transmit data through the CSMA part of the superframe. The other nodes will transmit in the TDMA part of the superframe. 

### 5.2. Simulation Results and Discussion

#### 5.2.1. Packet Delivery Ratio

**Figure 7:**
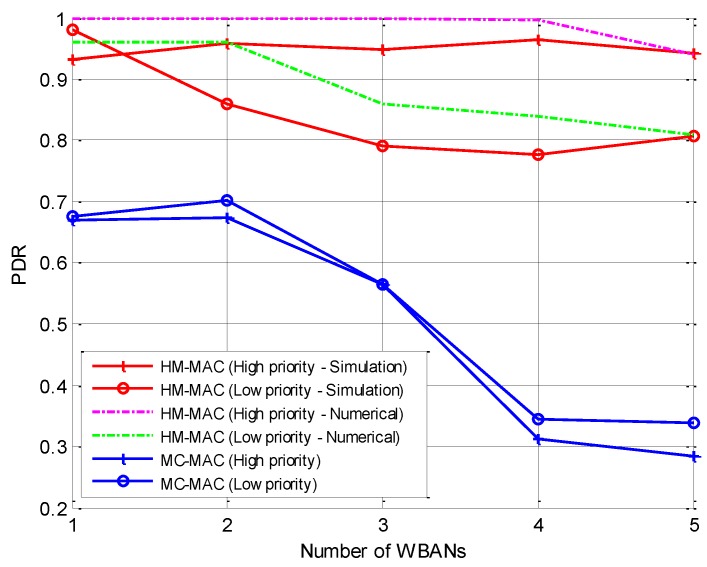
Packet delivery ratio.

Packet delivery ratio (PDR) is calculated as the number of successfully received data at the coordinator to the number of total sent packets at the sensor nodes. As shown in Figure 7, HM-MAC achieves higher PDR than MC-MAC. The simulation results are slightly lower than the expected numerical results because of the random deployment of multiple WBANs. It is noteworthy that, as shown in Table 1, the average channels for intra-WBAN is four while the number of total data channels is 11. Consequently, more than one WBAN needed to reuse the channel, resulting in a dropped packet at the coordinator. For high-priority traffic, HM-MAC achieves higher PDR owing to the proposed channel selection algorithm and CSMA/CA-based transmission. It is noted that any failed transmission will be retransmitted in the retransmitted CSMA/CA portion. Therefore, even for low-priority traffic, HM-MAC is better than MC-MAC.

#### 5.2.2. Average End-to-End Delay

**Figure 8:**
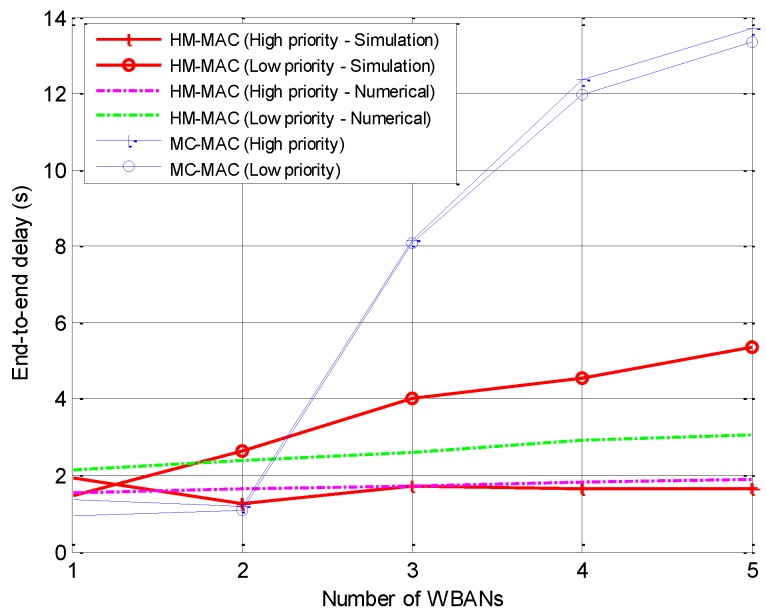
Average end-to-end delay.

The average end-to-end delay is calculated as the time when a packet is received at the coordinator to the generated time at the sensor nodes. The end-to-end delay consists of the duration of the CSMA/CA for high-priority traffic or the waiting time for low-priority traffic. Because of the high-density network and the reuse of idle channels, the sensor nodes need to perform CSMA/CA for a longer duration, which leads to a high end-to-end delay. For high-priority traffic, HM-MAC has a shorter end-to-end delay than MC-MAC, as shown in Figure 8. For high-priority traffic, the end-to-end delay of the simulation results is similar to that of the numerical results. For low-priority traffic, however, the end-to-end delay of the simulation results is higher than that of the numerical results due to the long waiting time of the CSMA part. The intra-WBAN transmission algorithm allows high-priority users to access the channel after receiving the beacon signal and to switch to other idle channels to ensure the continuity of transmission. Nevertheless, low-priority users have to wait until end of the CSMA/CA portion to transmit on the scheduled slot, which causes a higher delay.

#### 5.2.3. Throughput

**Figure 9:**
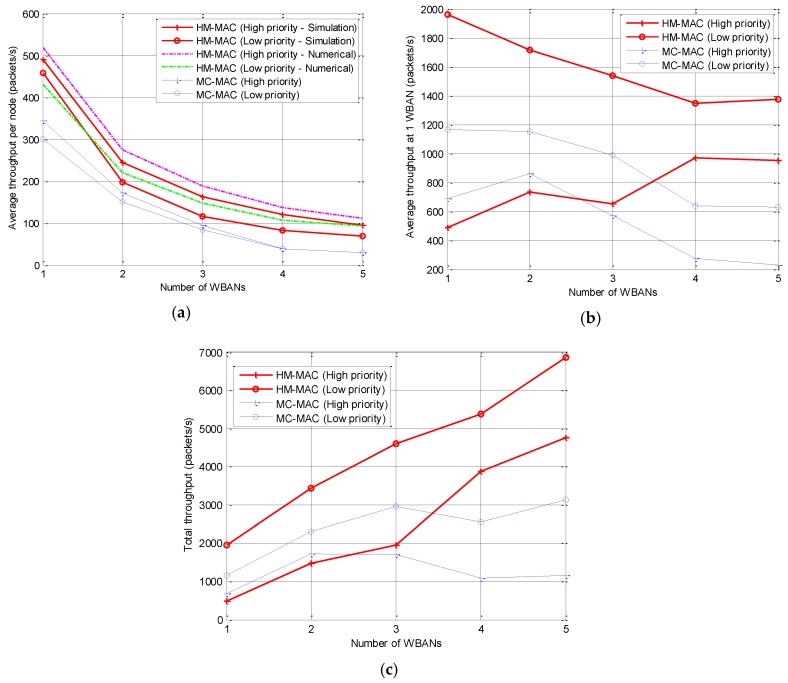
Network throughput: (**a**) average throughput per node; (**b**) average throughput per WBAN; (**c**) total throughput of the multi-WBAN network.

Throughput is the total successful received packets per total transmission time, which can be measured by packets per second. In HM-MAC, the high-priority node will send data in the CSMA part of the superframe; if the channel is busy, the node will switch to another channel. However, the low-priority nodes only transmit data in the TDMA part without switching the channel. In a dense environment, the WBAN may reuse the channel because of the limited resource of the channels. The average throughput per node is shown in Figure 9a; the successfully received packets at the coordinator decrease while the number of WBANs increase in both HM-MAC and MC-MAC. The simulation results give the lower average throughput than the numerical results, which are caused by the random packet length and the packet delivery ratio as shown in Figure 7. Nevertheless, the average throughput per node of the high-priority traffic is higher than that of the low-priority traffic in HM-MAC. In Figure 9b, the average throughput per WBAN is evaluated. To ensure the quality of the high-priority traffic in a multi-WBAN environment, the average throughput of the low-priority traffic decreases with the slight increase in the high-priority traffic. Because any failed transmissions will be retransmitted in the CSMA part, the higher priority will occupy the channel for transmitting while the low-priority nodes have to wait until the end of transmission. The total network throughput is shown in Figure 9c; HM-MAC achieves a higher network throughput than MC-MAC even though both algorithms show the increasing trend. However, the total throughput of the high-priority nodes is lower than that of the low-priority nodes; this can be explained as follows: The number of high-priority nodes is approximately 20% of the total nodes, and each node generates the same amount of packets to transmit to the coordinator. Therefore, the total generated packets of the higher-priority nodes is lower than that of the lower-priority nodes. 

#### 5.2.4. Energy Consumption

**Figure 10:**
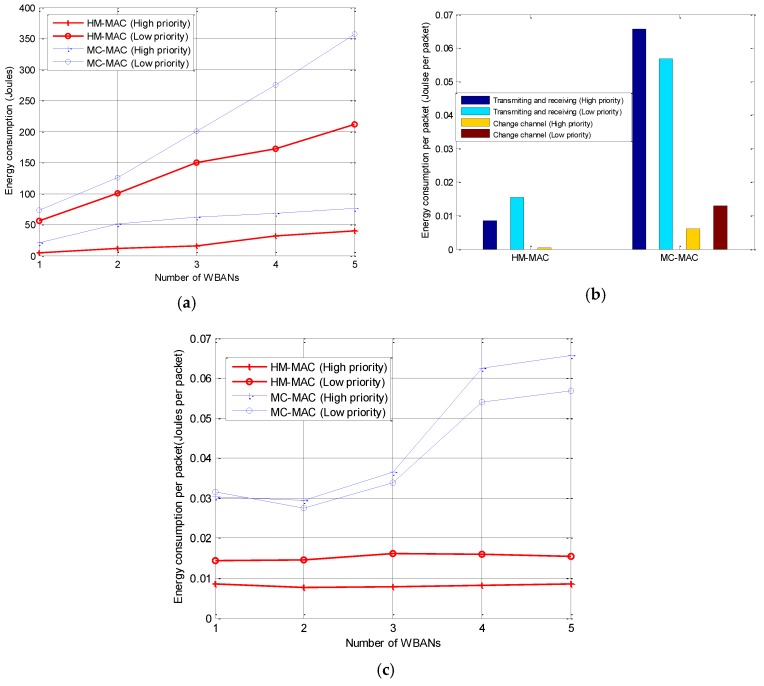
Energy consumption: (**a**) total energy consumption; (**b**) energy consumption per WBAN; (**c**) energy consumption per packet.

Energy consumption is calculated as the energy required for transmitting and receiving control packets, data packets, negotiation packets, and the energy for switching channels. However, we do not consider the energy consumption for the idle listening of nodes. The total energy consumption for transmitting and receiving increases with the number of WBANs, as shown in Figure 10a. The energy consumption for the transmission of higher-priority nodes is lower in both cases even though HM-MAC consumes less energy than MC-MAC. In Figure 10b, we consider the energy consumption of different tasks of both HM-MAC and MC-MAC. The energy consumed for transmitting and receiving high-priority traffic is lower than that of low-priority traffic in both cases. Nonetheless, the energy consumption for changing the channel of HM-MAC is lower compared to MC-MAC, because only the nodes with high-priority traffic can change the working channel. The nodes transmitting in TDMA do not change the working channel; therefore, energy is saved. However, in MC-MAC, nodes with different traffic priorities will occupy the channel using CSMA/CA with different backoff values while being able to switch the working channel to avoid collisions. Nonetheless, it may cause higher energy consumption in MC-MAC. In addition, the energy consumption per packet is calculated by the fraction of the total energy consumption to the successfully received packets. In Figure 10c, the average energy consumption per packet of HM-MAC is less than that of MC-MAC.

## 6. Conclusions

Herein, we proposed a hybrid multi-channel medium access control, called HM-MAC, for WBANs in order to successfully mitigate inter-WBAN interference. In HM-MAC, a superframe consists of the random access CSMA/CA phase and the scheduled access TDMA phase. The CSMA/CA phase allows the higher-priority users to transmit data packets with low latency and high reliability, whereas the TDMA phase enables the periodic data to be transmitted with no contention and high reliability. The channel selection algorithm allows the coordinators to select the channels for intra-WBAN transmission to adapt to the priority traffic conditions, by which the collision between neighboring WBANs is avoided. In addition, HM-MAC consumes less energy compared to the conventional protocol. The performance study shows that the HM-MAC protocol achieves higher network performance with lower energy consumption and lower delay than the conventional protocol.

In HM-MAC, the negotiation between WBANs is the necessary step that helps the coordinator to learn about the network and environment. Therefore, the number of control packets in the network will be increased, which may result in high energy consumption and long waiting time at each WBAN. As future work, we plan to investigate the energy efficiency of multi-channel MAC protocols without negotiations in multi-WBAN scenarios.

## Figures and Tables

**Table 1:** Simulation parameters.

Parameters	Value
Number of WBANs	1–5
Number of sensors per WBAN	10
Traffic priority	20% high priority (Carrier sense multiple access – CSMA) 80% low priority (Time division multiple access – TDMA)
Number of channels per WBAN	4
Number of channels in the network	12 channels
Simulation area	10 m × 10 m
Transmission range	2 m
Distance between coordinator and nodes	0.6–1.4 m
Data rate	250 kbps
Packet length	50–150 bytes (depends on the traffic at the sensor nodes)
Beacon size	15 bytes
Negotiation size	5 bytes
Superframe length	100 ms
Channel clear assessment time	0.01 ms
Number of TDMA slots per superframe	4–6 slots
Frequency	2.4 GHz
Traffic arrival rate	10 packets per second
Transmit current [25]	17.4 mA
Receive current [25]	19.7 mA
Energy consumption per each channel switching [25]	2 mJ
Voltage	3.3 V
